# MCU knockdown attenuates cisplatin-induced damage of auditory cells: potential involvement of PI3K/AKT/mTOR signaling and autophagy-related processes

**DOI:** 10.3389/fphar.2026.1913816

**Published:** 2026-08-19

**Authors:** Yue Xu, Yang Li, Jie Mao, Jia Xue, Demin Zhu, Yanan Li, Hao Zhao, Jianfeng Li, Huiming Yang, Rongjun Man

**Affiliations:** 1 Department of Otolaryngology-Head and Neck Surgery, Shandong Provincial Hospital, Shandong University, Jinan, Shandong, China; 2 Department of Otolaryngology-Head and Neck Surgery, Shandong Provincial Hospital Affiliated to Shandong First Medical University, Jinan, Shandong, China; 3 Shandong Second Provincial General Hospital, Jinan, Shandong, China; 4 Department of Otolaryngology, Head and Neck Surgery, People’s Hospital, Peking University, Beijing, China

**Keywords:** autophagy, hearing loss, MCU, ototoxicity, PI3K/Akt/mTOR pathway

## Abstract

**Introduction:**

The mitochondrial calcium uniporter (MCU) mediates mitochondrial Ca^2⁺^ uptake, but its involvement in cisplatin-induced ototoxicity remains poorly understood. We investigated MCU expression and its association with cisplatin-induced injury in mouse cochlear basilar membranes and HEI-OC1 cells.

**Methods:**

MCU protein abundance and localization were assessed by Western blotting and immunofluorescence. MCU expression was genetically reduced by lentiviral knockdown, and Ru360 was used as a pharmacological intervention. Apoptosis-related proteins and transcriptomic changes were evaluated. An acute cisplatin-induced ototoxicity model was established in C57BL/6 mice and assessed using auditory brainstem response (ABR) measurements and outer hair cell counts.

**Results:**

Cisplatin increased MCU expression and reduced SERCA2 expression; the cisplatin-induced increase in MCU was partially reversed by BAPTA-AM and the endoplasmic reticulum stress inhibitor 4-PBA. MCU knockdown did not affect basal cell viability but attenuated cisplatin-induced reductions in viability, TUNEL positivity, and cleaved caspase-3. It was also associated with increased SERCA2 and decreased IP3R, GRP75, and VDAC1 expression. RNA sequencing identified differentially expressed genes enriched in autophagy-related processes and mTOR signaling. MCU knockdown was associated with increased PI3K/AKT/mTOR signaling and changes in Beclin1, LC3B-II, and p62. Ru360 cotreatment partially reproduced the protective phenotype *in vitro* and was associated with attenuated ABR threshold elevation at selected frequencies and reduced outer hair cell loss *in vivo*.

**Discussion:**

These findings support an association between MCU-related signaling, PI3K/AKT/mTOR pathway activation, altered autophagy-related markers, and reduced cisplatin-induced cochlear injury.

## 1 Introduction

Hearing loss, one of the most urgent unmet health needs, affects over 1.5 billion people worldwide and continues to rise, severely impairing daily life and imposing a heavy societal burden (Chadha et al., 2021; Haile et al., 2021). Sensorineural hearing loss (SNHL), one of the most prevalent forms of deafness, causes permanent, irreversible hearing loss through hair cell death and arises from aging, noise, and ototoxic drugs (Cunningham and Tucci, 2017). Among these, cisplatin has been identified as one of the most common agents causing SNHL (Gersten et al., 2020; Pienkowski, 2021).

Cisplatin, a first-line antitumor chemotherapeutic drug, is widely used to treat multiple solid tumors (Ghosh, 2019). Despite the therapeutic value of eliminating tumors, the side effects of cisplatin cannot be ignored (Kros and Steyger, 2019). Hearing loss develops in 23%–50% of adults and up to 60% of children exposed to cisplatin (Moke et al., 2021). The pathogenesis of cisplatin-induced hearing loss has been found to be closely related to the generation of reactive oxygen species (ROS), mitochondrial dysfunction, autophagy, and disturbances in calcium homeostasis (Fetoni et al., 2022). Inner ear structures, such as the cochlear basilar membrane, spiral ganglion, and stria vascularis, are affected by these events (Breglio et al., 2017). Although cisplatin ototoxicity has been studied for decades, its precise molecular mechanisms remain incompletely understood (Yang et al., 2023).

Previously, we found that cisplatin disrupts calcium homeostasis and triggers endoplasmic reticulum (ER) stress, leading to apoptosis (Xu et al., 2024; Zhao et al., 2022). Calcium, a key second messenger, plays pivotal roles in biological processes such as proliferation, survival, and immune responses (Kong et al., 2021). Its stability relies not only on calcium channels in the plasma membrane and ER but also on mitochondria (Gottlieb and Bernstein, 2016). Indeed, mitochondria can take up calcium released from the ER when the ER is stimulated under certain physiological or pathological conditions. The intracellular calcium-buffering function of mitochondria depends on specific channels (Xu et al., 2018).

Of these channels, mitochondrial calcium uniporter (MCU), a 40-kDa protein encoded by the MCU gene (De Stefani et al., 2011), is a highly selective calcium uptake channel located in the inner mitochondrial membrane (IMM). MCU has been under intensive focus regarding the function of regulating mitochondrial homeostasis since it was first identified as the pore-forming molecule of the MCU complex (Baughman et al., 2011). Besides MCU, mitochondrial calcium uptake proteins 1 and 2 (MICU1 and MICU2), the essential MCU regulator (EMRE), the mitochondrial calcium uniporter beta subunit (MCUb), and mitochondrial calcium uniporter regulator 1 (MCUR1) together compose the complex (Yoo et al., 2018). Accumulating evidence indicates that MCU is intimately connected with diverse diseases, including cancer, cardiovascular and cerebrovascular disease, neurodegenerative disease, and neuromuscular disorders (Calvo-Rodriguez et al., 2020; Li et al., 2021; Zeng et al., 2018). Importantly, systemic deletion of the MCU gene causes lethality in neonatal mice but results in impairment of cardiac function and neural signal transduction rather than death in adult mice, thereby implying that the MCU gene exhibits distinct roles between developmental stages (Ashrafi et al., 2020; Liu et al., 2021; Patron et al., 2018). Additional experiments have shown that overexpression of MCU enhances cell apoptosis induced by various stimuli, revealing the important role of MCU-dependent mitochondrial calcium uptake in apoptosis (Liao et al., 2015).

Even though the diverse functions of MCU have been revealed mainly in cerebrovascular, myocardial, and skeletal muscle tissues, the effects of MCU on the mouse cochlea in response to exogenous stress have been explored only sparingly (Wang et al., 2019). However, whether MCU regulates cisplatin-induced ototoxicity has not been studied to date. Therefore, the present study investigated the expression of MCU in the cochlea of C57BL/6 mice and, using genetic knockdown and pharmacological inhibition, examined how MCU contributes to cisplatin-induced death of cochlear hair cells and HEI-OC1 cells, with a focus on calcium homeostasis and PI3K/AKT/mTOR-mediated suppression of autophagy.

## 2 Materials and methods

### 2.1 Animals and treatments

All animal experimental methods were approved by the Animal Care Committee of Shandong University, Jinan, China (NO. ECAESDUSM 20123011). Postnatal day 4 C57BL/6 mice were purchased from Spelford (Beijing) Biotechnology Company. After decapitation, the inner ears were removed from temporal bones in cold phosphate-buffered saline (PBS). The stria vascularis, spiral ligament, Reissner’s membrane, and tectorial membrane were gently cut off without causing damage to hair cells under a stereomicroscope. Then, obtained basilar membranes were attached to 48-well round coverslips covered with Cell-Tak and cultured in the incubator at 37 °C, 5% CO_2_ for subsequent experiments. For the *in vivo* experiments, two-month-old male C57BL/6 mice were kept under specific pathogen-free (SPF) conditions at 25 °C, 40%–50% humidity, with a 12 h light/dark cycle and free access to standard food and water.

### 2.2 Cell culture

The House Ear Institute-Organ of Corti 1 (HEI-OC1) cells, a commonly used model cell line for auditory research, were cultured in dishes in high-glucose DMEM supplemented with 10% fetal bovine serum in an incubator with 10% CO_2_, at 33 °C. No antibiotic was used during cell culture.

### 2.3 Western blotting (WB)

Cells and cochlear basilar membrane subjected to different stimuli were harvested with RIPA cocktail containing phenylmethylsulfonyl fluoride (PMSF; Servicebio) and phosphatase inhibitors (Servicebio). The supernatant was then collected after centrifugation for 15 min at 16,000 rpm and 4 °C. A BCA protein assay kit was used to determine the concentration of the protein samples, which were then denatured by adding loading buffer and boiling for 10 min at 100 °C. Next, the samples were loaded onto and separated by 6%–12% SDS-PAGE according to the target proteins, then transferred to polyvinylidene fluoride (PVDF) membranes (Merck Millipore). The PVDF membranes were incubated with diluted primary antibodies at 4 °C overnight after blocking by 5% skim milk at room temperature for 1 h. Then, the membranes were incubated with the secondary antibodies at room temperature for 1 h. Eventually, the results were detected using an ECL kit (Millipore) and analyzed by ImageJ software. The following primary antibodies were used: anti-MCU (14,997, Cell Signaling Technology [CST]), anti-cleaved caspase-3 (9664, CST), anti-glucose-regulated protein 78 (GRP78; 21,685, Abcam), anti-SERCA2 (150,435, Abcam), anti-inositol 1,4,5-trisphosphate receptor (IP3R; 108,517, Abcam), anti-glucose-regulated protein 75 (GRP75; 3593, CST), anti-voltage-dependent anion channel 1 (VDAC1; GB111939-100, Servicebio), anti-B-cell lymphoma 2 (Bcl-2; 196,495, Abcam), anti-phosphorylated phosphoinositide 3-kinase (p-PI3K; 182,651, Abcam), anti-3-phosphoinositide-dependent protein kinase 1 (PDK1; A1665, Abclonal), anti-phosphorylated protein kinase B (p-AKT; 81,283, Abcam), anti-AKT (4691, CST), anti-phosphorylated mechanistic target of rapamycin (p-mTOR; 5536, CST), anti-sequestosome 1 (p62; 109,012, Abcam), anti-Beclin1 (3495, CST), anti-microtubule-associated protein 1 light chain 3 beta (LC3B; 83,506, CST), anti-glyceraldehyde-3-phosphate dehydrogenase (GAPDH; 60,004-1, Proteintech), anti-β-actin (AC026, ABclonal). MCU was detected as a single band at an apparent molecular weight of ∼30 kDa, consistent with the mature protein following cleavage of its N-terminal mitochondrial targeting sequence, whereas the full-length precursor is ∼40 kDa.

### 2.4 Drug treatments

It has been validated in our previous study that cisplatin (Sigma-Aldrich, United States) administration at a concentration of 30 μM for 24 h achieved the median lethal dose (LD50) in both HEI-OC1 cells and mouse cochlear basilar membranes (Yang et al., 2018). The membrane-permeable intracellular Ca^2+^ chelator BAPTA-AM (MedChemExpress, United States) was used for blocking cytoplasmic calcium with a concentration of 30 μM. 4-phenylbutyric acid (4-PBA; MedChemExpress, United States) was used for suppressing ER stress with a concentration of 5 mM. Ru360 (Santa Cruz, United States) was used for inhibiting MCU expression with a concentration of 10 μM in HEI-OC1 cells. For *in vivo* administration, cisplatin was dissolved in 0.9% normal saline to a working concentration of 1 mM (0.3 mg/mL) with gentle warming at 37 °C and protected from light. Ru360 was initially dissolved in sterile water to prepare a 1 mM stock solution, which was further diluted 200-fold with 0.9% normal saline to a final working concentration of 5 μM immediately before use. After all mice were randomly divided into three groups, namely the Control group, the cisplatin (Cis) group, and the Ru360 + Cis group, with six mice in each group, they were intraperitoneally injected at a standardized volume of 10 mL/kg body weight. Specifically, the Control group received an equivalent volume of vehicle, the Cis group received cisplatin at 10 μmol/kg, and the Ru360 + Cis group received a combined injection solution containing Ru360 at 50 nmol/kg and cisplatin at 10 μmol/kg. All injection solutions were freshly prepared before administration.

### 2.5 Immunofluorescence

Cells and cochlear basilar membrane subjected to different stimuli were collected and washed with cold PBS, then fixed with 4% paraformaldehyde (PFA, Biosharp) for 10 and 30 min at room temperature, respectively. After fixation, 0.3% Triton X-100 was used for permeabilization, and 1% bovine serum albumin (BSA) was used for blocking at room temperature. The materials were incubated with primary antibodies at 4 °C overnight and then, after being rinsed three times with PBS, incubated with the corresponding secondary antibodies for 1 h at room temperature. Immunofluorescence images of postnatal day 4 and adult mouse cochlear basilar membrane whole mounts and HEI-OC1 cells were acquired using a TCS SP8 laser-scanning confocal microscope (Leica Microsystems, Wetzlar, Germany). Images were captured using 40×, 63×, or ×100 objectives, depending on the specimen type and experimental purpose. The 405-, 488-, and 647-nm laser lines were used to excite the corresponding fluorophores. Image acquisition was controlled using LAS X software (Leica Microsystems). For comparisons among experimental groups within the same experiment, images were acquired using consistent imaging settings. The acquired images were processed and analyzed using ImageJ software. The following primary antibodies were used: anti-MCU (14,997, CST), anti-LC3B (83,506, CST), anti-Myosin 7a (MYO7A 138-1, DSHB; 256,790, Proteus-biosciences), anti-cleaved caspase-3 (9664, CST).

### 2.6 Terminal deoxynucleotidyl transferase-mediated deoxyuridine triphosphate nick-end labeling (TUNEL) assay

The apoptosis status of HEI-OC1 cells and hair cells was detected by TUNEL staining using One Step TUNEL Apoptosis Assay Kit (Red Fluorescence) (Meilunbio). Cells and cochlear basilar membranes exposed to various stimuli were fixed with 4% PFA for 30 min at room temperature and then rinsed 3 times with PBS. 0.3% Triton X-100 was used to permeabilize cells and cochlear basilar membrane, and 1% BSA was used for blocking. Subsequently, samples were incubated with a TUNEL working solution configured according to the reagent instructions at 37 °C for 1 h protected from light. The cell nuclei were stained with DAPI. A laser scanning confocal microscope (TCS SP8, Leica) was used for image capture.

### 2.7 Lentivirus infection

MCU knockdown lentivirus was designed and constructed by GeneChem (Shanghai, China). HEI-OC1 cells were seeded in a 6-well plate at a density of 3 × 10^4^/mL for 24 h before infection. Based on preliminary experiments, a multiplicity of infection (MOI) of 50 was used. Optimal volumes of control lentivirus and MCU knockdown lentivirus were added to the two groups receiving control short hairpin RNA (shControl) and MCU-targeting short hairpin RNA (shMCU), respectively. To obtain a stably infected cell line, puromycin at a concentration of 5 μg/mL was added to the complete medium to eliminate noninfected cells after 72 h of infection. The knockdown efficiency was evaluated by WB.

### 2.8 Cell viability assay

Cells were seeded in a 96-well plate and cultured in an incubator for 24 h before drug administration. After stimuli, a CCK-8 kit (Dojindo, Japan) was used for the cell viability test according to the instructions. In brief, samples were incubated with 10 μL working solution per well for about 1 h at 37 °C, and then the optical density (OD) value was measured with a microplate reader (SpectraMax M2, United States) at a wavelength of 450 nm.

### 2.9 Mito-Tracker staining

The mitochondria were detected by Mito-Tracker deep red FM (Beyotime, China) following the provided instructions. The drug-treated HEI-OC1 cells were collected and washed three times with PBS, then incubated with Mito-Tracker deep red FM working solution diluted with DMEM to a final concentration of 200 nM for 30 min at 37 °C protected from light. Cell nuclei were counterstained with Hoechst 33,258 (Yeasen, China) at a concentration of 5 μg/mL. The final fluorescence images were captured by an inverted fluorescent microscope (Olympus, Japan).

### 2.10 RNA sequencing analysis

Total RNA was extracted from HEI-OC1 cells using TRIzol reagent. RNA purity and concentration were assessed using a NanoDrop 2000 spectrophotometer, and RNA integrity was evaluated using an Agilent 2100 Bioanalyzer/LabChip GX system. RNA sequencing was performed on 12 samples from four experimental groups (shControl, shMCU, shControl + Cis, and shMCU + Cis), with three independent biological replicates per group. The transcriptomic analysis presented in this study focused on the comparison between the shControl + Cis and shMCU + Cis groups, with three independent biological replicates per group. Libraries passing quality control were sequenced on an Illumina NovaSeq 6000 platform using a 150-bp paired-end sequencing strategy. For the six samples included in this comparison, 45.70–47.42 million raw reads were generated per sample. After quality filtering, 43.88–45.64 million clean reads, corresponding to 6.58–6.85 Gb of clean sequence data, were retained per sample. The mean Q20 and Q30 values were 98.95% and 97.10%, respectively. Genes with a fold change greater than 1.5 or less than 1/1.5, corresponding to an absolute log2 fold change greater than 0.585, and an FDR-adjusted P value of less than 0.05 were considered differentially expressed.

### 2.11 Auditory brainstem response (ABR) measurement

ABR measurements were performed twice per mouse, before treatment and 3 days after completion of treatment, using the TDT System III (Tucker Davis Technologies, United States). Under the anesthesia condition, the mice were placed in a soundproof chamber, with the recording, reference, and grounding electrodes fixed to their cranial vertex, postauricular mastoid, and the middle line of their back, respectively. Tests were conducted at frequencies of 4, 8, 12, 16, 24, and 32 kHz, in which the sound intensity was reduced from 90 dB to 20 dB in 10 dB decrements. After measurements, the mice were delicately transferred to a 37 °C-heating pad until full consciousness was regained.

### 2.12 Statistical analyses

GraphPad Prism Version 8.3 (GraphPad Software, San Diego, CA, United States) was used for all statistical analyses. Data were shown as mean ± SD, and each group included at least three independent biological replicates. Student’s t-test or one-way analysis of variance (ANOVA) was used for comparisons involving a single independent factor, as appropriate. ABR and Body-weight data were analyzed using two-way ANOVA. Tukey’s multiple-comparisons test was performed for pairwise comparisons among the treatment groups at each time point or stimulus frequency. A value of P < 0.05 was considered statistically significant.

## 3 Results

### 3.1 MCU expression level was elevated by cisplatin exposure

**FIGURE 1:**
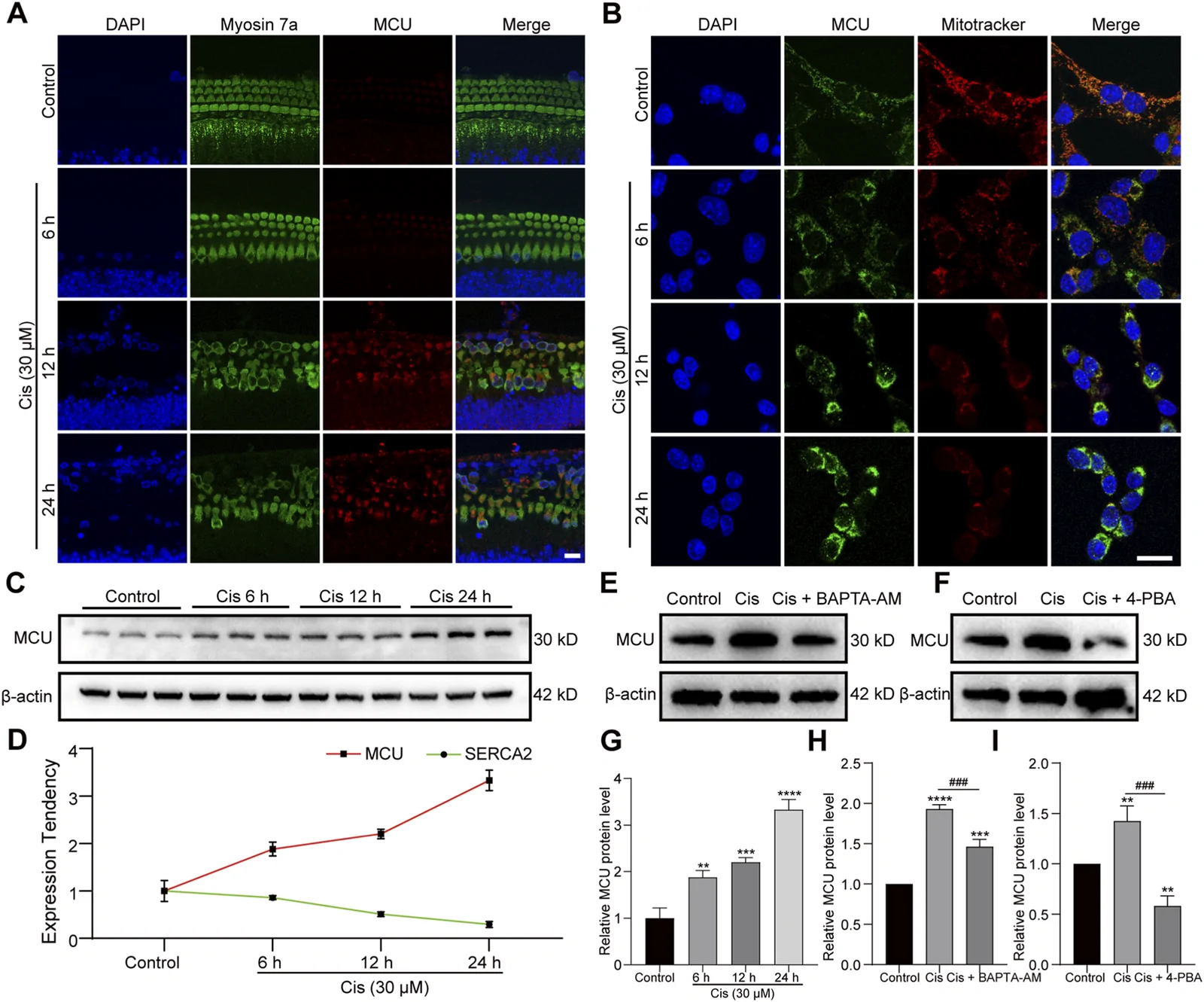
MCU expression level was elevated by cisplatin exposure. **(A)** Immunofluorescence staining was performed to detect changes in MCU expression in the basilar membrane after cisplatin treatment. **(B)** Co-staining of MCU immunofluorescence and Mito-Tracker in HEI-OC1 cells was conducted to observe the specific localization of MCU. **(C)** WB analysis was used to assess the expression changes of MCU in the basilar membrane at different time points of cisplatin exposure. **(D)** A line chart illustrated the trend of MCU and SERCA2 expression changes. **(E)** WB results showed partial recovery of MCU expression levels after 30 μM BAPTA-AM application in HEI-OC1 cells. **(F)** WB showed a significant decrease in MCU expression after 5 mM 4-PBA treatment in HEI-OC1 cells. **(G)** Quantification of the WB results shown in panel **(C)**. **(H)** Quantification of the WB results shown in panel **(E)**. **(I)** Quantification of the WB results shown in panel **(F)**. *p < 0.05, **p < 0.01, ***p < 0.001, ****p < 0.0001 compared to the control group. n = 3. Scale bar for the cochlear basilar membrane = 25 μm. Scale bar for HEI-OC1 cells = 30 μm.

The immunofluorescence results for the cochlear basilar membrane showed that MCU was expressed in both the inner and outer hair cells, and that its expression level was significantly upregulated after 12 h and 24 h of cisplatin exposure (Figure 1A). To verify the exact localization of MCU, Mito-Tracker staining was performed in HEI-OC1 cells; the results revealed that MCU co-localized with mitochondria and showed an expression pattern similar to that in hair cells under cisplatin stimulation. Unlike MCU, the immunofluorescence intensity of Mito-Tracker gradually diminished (Figure 1B). WB results showed the same trend as the immunofluorescence results (Figures 1C,G). Interestingly, the dynamic expression trends of MCU and SERCA2 showed an opposite pattern following cisplatin exposure. MCU expression gradually increased, whereas SERCA2 expression exhibited a declining trend (Figure 1D). Next, we chose calcium ion chelator BAPTA-AM and ER stress inhibitor 4-PBA as tools to investigate the factors that influence MCU expression. The results showed that the cisplatin-induced elevated expression level of MCU was reversed by BAPTA-AM and 4-PBA (Figures 1E,F,H,I).

### 3.2 Cisplatin altered autophagy-related markers in cochlear basilar membranes

**FIGURE 2:**
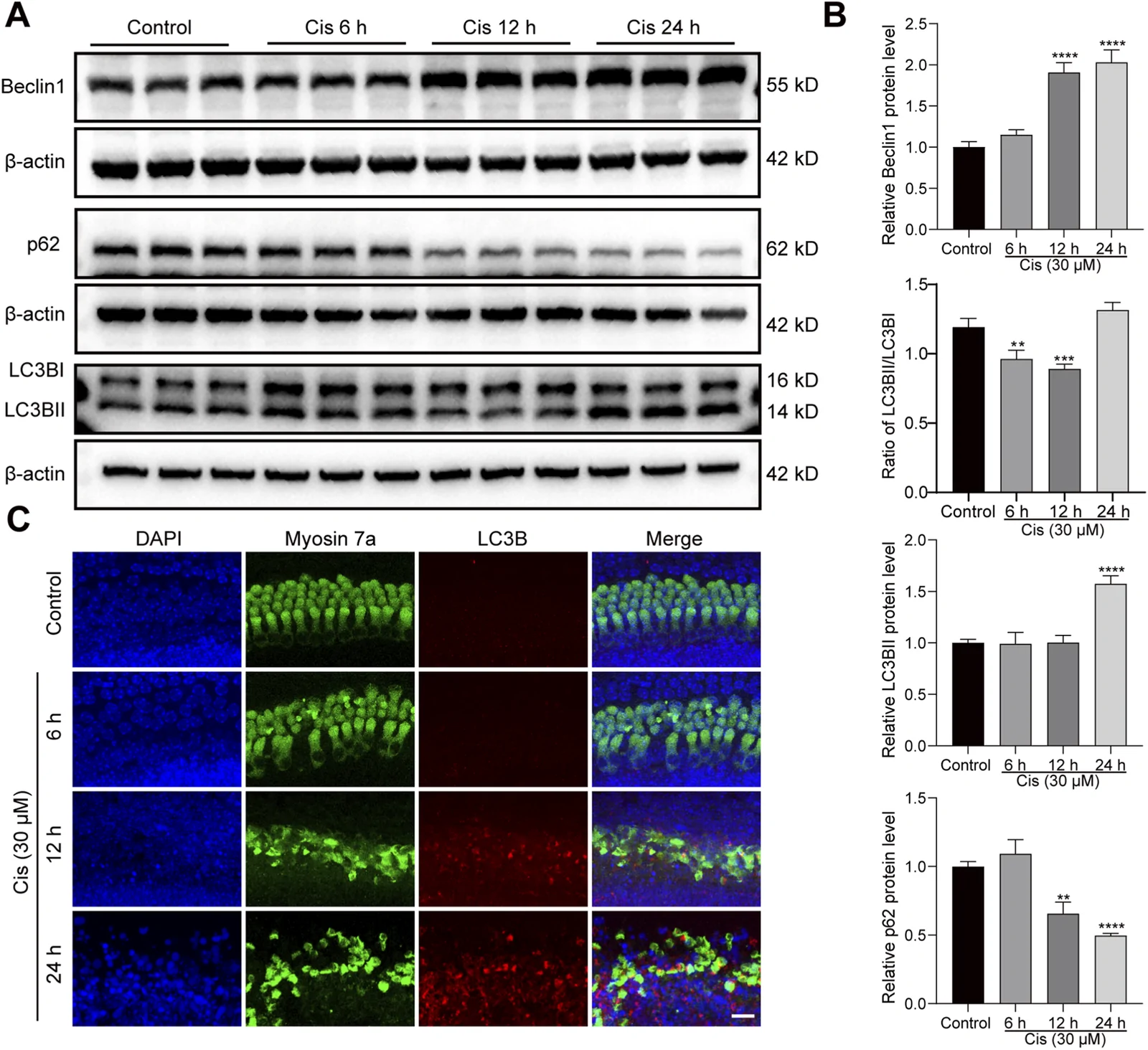
Cisplatin induced the increase of cell autophagy. **(A)** WB analysis was conducted to examine the changes in autophagy levels after cisplatin treatment in the basilar membrane. The results indicated an increase in the expression of Beclin1 and LC3B-II, accompanied by a decrease in p62 expression under the influence of cisplatin. **(B)** Quantification of the WB results shown in panel **(A)**. **p < 0.01, ***p < 0.001, ****p < 0.0001 compared to the Control group. n = 3. **(C)** Immunofluorescence staining was used to detect changes in LC3B expression in the basilar membrane after cisplatin treatment.

Next, we examined whether cisplatin treatment induced autophagy in cochlear basilar membranes by assessing the expression of Beclin1, LC3B-II, and p62/SQSTM1, three commonly used autophagy-related markers reflecting autophagy initiation, autophagosome formation, and autophagic substrate degradation, respectively. WB results revealed that Beclin1 was markedly increased after cisplatin exposure for 12 h. The LC3B-II level normalized to the loading control remained unchanged at 6 and 12 h but was significantly increased after 24 h of cisplatin exposure. In contrast, the LC3B-II/LC3B-I ratio was significantly decreased at 6 and 12 h compared with the control group and subsequently increased at 24 h compared with the 6- and 12-h groups, although it was not significantly different from the control level. In contrast, p62 expression was reduced under cisplatin insult (Figures 2A,B). In addition, the immunofluorescence results showed that the fluorescence intensity of LC3B was increased compared to the control group with cisplatin administration (Figure 2C).

### 3.3 MCU knockdown alleviated cisplatin-induced damage and changed expression levels of related calcium ion channels

**FIGURE 3:**
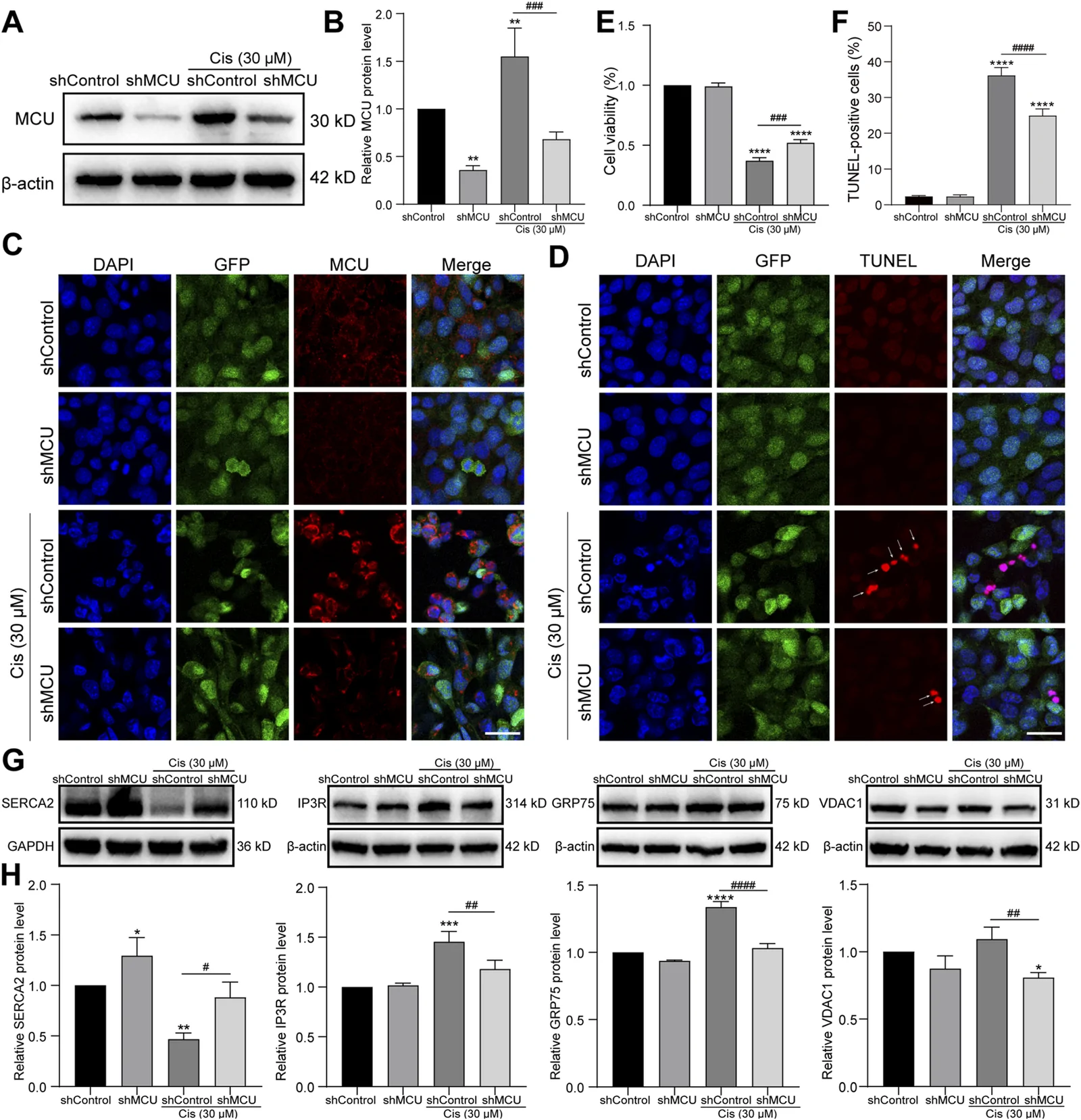
MCU knockdown mitigated cisplatin-induced cell damage. **(A,B)** WB analysis was conducted to assess the knockdown efficiency of MCU in HEI-OC1 cells. **(C)** Immunofluorescence staining was used to verify the MCU knockdown efficiency. **(D)** TUNEL assay was employed to detect changes in the number of apoptotic cells after MCU knockdown. **(E)** CCK-8 assay was performed to observe alterations in cell survival rate following MCU knockdown. **(F)** Quantification of TUNEL-positive cells in panel **(D)**, expressed as a percentage of total cells. **(G,H)** WB was utilized to examine expression changes of other related Ca^2+^-handling proteins, namely SERCA2, IP3R, GRP75, and VDAC1, after MCU knockdown in HEI-OC1 cells. Statistical significance is denoted as *p < 0.05, **p < 0.01, ***p < 0.001, and ****p < 0.0001 compared to the Control group. ^#^p < 0.05, ^##^p < 0.01, ^###^p < 0.001, and ^####^p < 0.0001 compared to the shControl + Cis group. n = 3. The scale bar for HEI-OC1 cells represents 30 μm.

To further explore MCU’s roles in cisplatin ototoxicity, we carried out the MCU knockdown experiment via lentivirus infection on HEI-OC1 cells. The knockdown efficiency was evaluated by WB. Compared with the control group, the MCU expression level was significantly decreased in the knockdown group (Figures 3A,B). Immunofluorescence results were consistent with WB (Figure 3C). Subsequently, a TUNEL assay was conducted to observe cell death following MCU knockdown, and the result showed there was no difference between the shControl group and the shMCU group. However, in the shMCU + Cis group, the ratio of TUNEL-positive cells was markedly reduced compared with the shControl + Cis group (Figures 3D,F). Consistent with this finding, the CCK-8 assay showed significantly higher cell viability in the shMCU + Cis group than in the shControl + Cis group, demonstrating that MCU knockdown alleviated the cisplatin-induced reduction in cell viability (Figure 3E). Changes in other related Ca^2+^-handling proteins were monitored by WB. The results showed that SERCA2 was markedly upregulated following MCU knockdown, and this increase persisted with cisplatin exposure. IP3R, GRP75, and VDAC1 showed the opposite change to SERCA2; that is, the expression of these Ca^2+^-handling proteins decreased after MCU knockdown under cisplatin administration (Figures 3G,H).

### 3.4 RNA-seq analysis of MCU knockdown revealed related possible genes and signaling pathways

**FIGURE 4:**
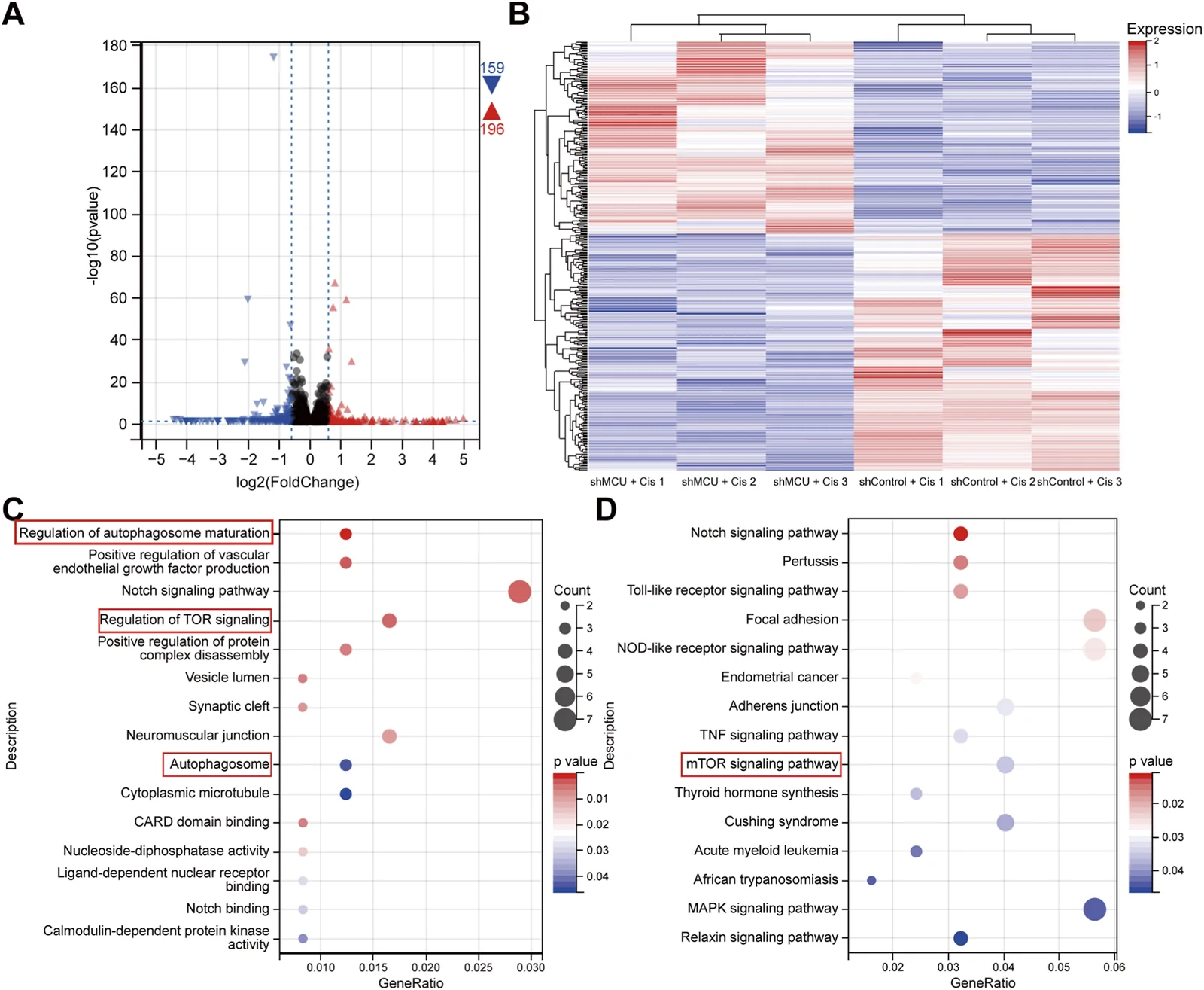
RNA-seq results revealed key genes and signaling pathways under the condition of MCU knockdown. **(A)** Differentially expressed genes were presented in the form of a volcano plot, with screening criteria set at fold change = 1.5 and p < 0.05. **(B)** Heatmap of differentially expressed genes. **(C)** GO analysis diagram. **(D)** KEGG pathway enrichment map.

To gain insight into the mechanisms by which MCU knockdown protects against cisplatin damage, we performed RNA sequencing to identify genes involved in this process, with or without MCU knockdown, in HEI-OC1 cells. The volcano plot showed the global differentially expressed genes (DEGs) between the shControl + Cis and shMCU + Cis groups. Compared with the shControl + Cis group, a total of 355 genes (196 upregulated and 159 downregulated) were markedly altered in the shMCU + Cis group (Figure 4A). A heatmap of the DEGs in the two groups is shown in Figure 4B. Gene Ontology (GO) analysis revealed prominent enrichment of the DEGs in diverse biological processes, including regulation of autophagosome maturation and regulation of target of rapamycin (TOR). In addition, in the cellular-component category, the vesicle lumen and autophagosome were enriched (Figure 4C). Kyoto Encyclopedia of Genes and Genomes (KEGG) analysis showed that the DEGs were enriched in several signaling pathways, including the mitogen-activated protein kinase (MAPK), Notch, and mTOR signaling pathways (Figure 4D). Given the autophagy- and TOR-related terms identified by GO analysis, we focused on the mTOR signaling pathway for subsequent validation.

### 3.5 MCU knockdown-mediated protection against cisplatin exposure was associated with activation of the PI3K/AKT/mTOR pathway

**FIGURE 5:**
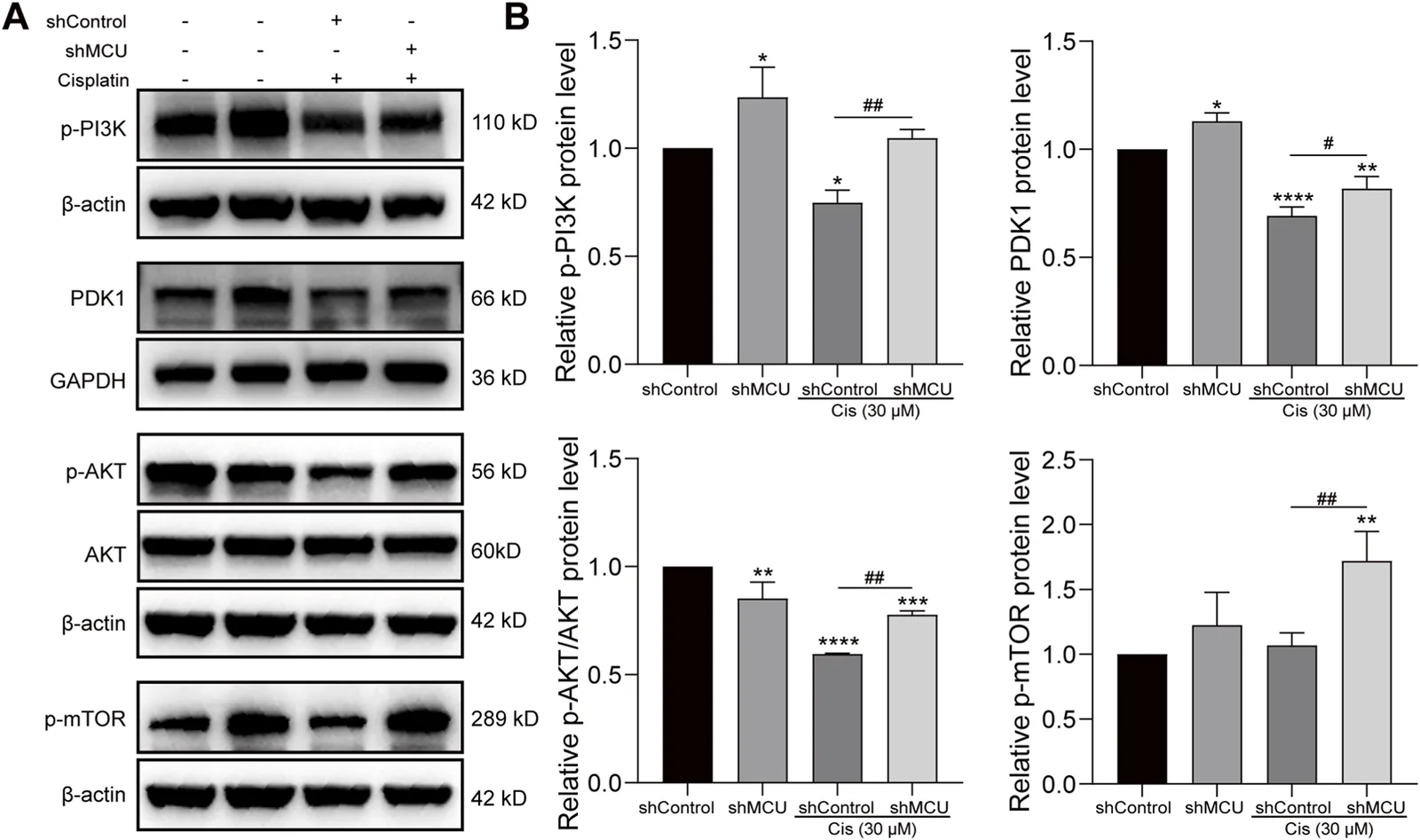
MCU knockdown activated the PI3K/AKT/mTOR pathway. **(A)** WB analysis was conducted to detect the expression changes of key factors in the PI3K/AKT/mTOR pathway after MCU knockdown in HEI-OC1 cells. **(B)** Quantification of the WB results shown in panel **(A)**. *p < 0.05, **p < 0.01, ***p < 0.001, ****p < 0.0001 compared to the shControl group. ^#^p < 0.05, ^##^p < 0.01 compared to the shControl + Cis group. n = 3.

Based on the RNA-seq results, the expression and/or phosphorylation states of key proteins involved in the PI3K/AKT/mTOR pathway were examined by WB. Compared with the shControl + Cis group, the levels of p-PI3K, PDK1, p-AKT, and p-mTOR were significantly increased in the shMCU + Cis group (Figure 5).

### 3.6 MCU knockdown altered autophagy-related markers and reduced cell apoptosis

**FIGURE 6:**
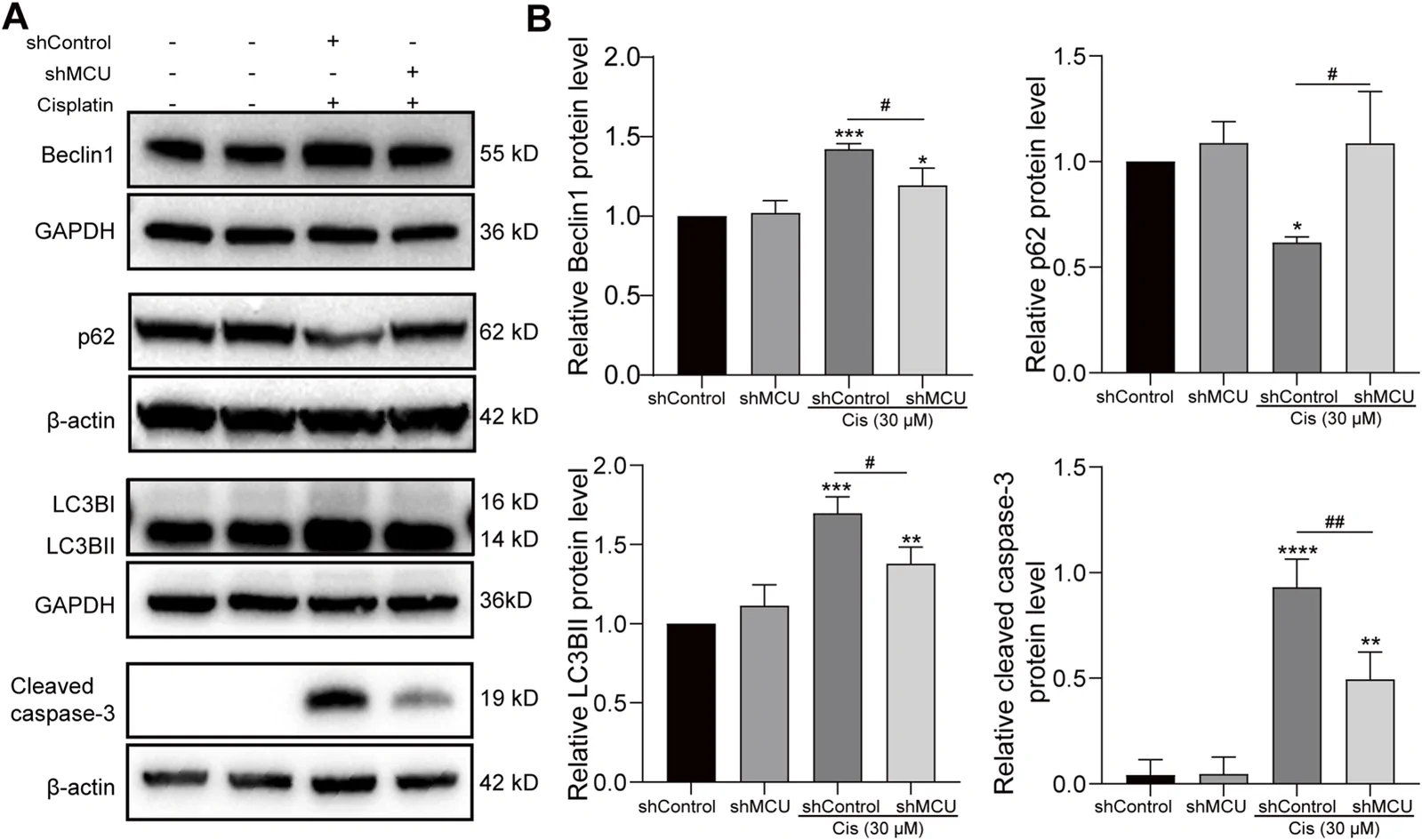
MCU knockdown inhibited autophagy. **(A)** WB analysis was conducted to detect changes in the expression of key autophagy factors in HEI-OC1 cells after MCU knockdown. **(B)** Quantification of the WB results shown in panel **(A)**. *p < 0.05, **p < 0.01, ***p < 0.001, ****p < 0.0001 compared to the Control group. ^#^p < 0.05, ^##^p < 0.01 compared to the shControl + Cis group. n = 3.

Next, we examined the expression of key autophagy-related proteins given the association between autophagy-related processes and PI3K/AKT/mTOR signaling. WB results showed that, compared with the untreated shControl group, the expression levels of Beclin1 and LC3B-II were markedly elevated, whereas p62 expression was decreased, in shControl + Cis group. However, within the cisplatin-treated groups, MCU knockdown significantly decreased Beclin1 and LC3B-II expression while increasing p62 expression compared with the shControl + Cis group. In addition, cleaved caspase-3, the critical executioner of apoptosis, was measured and was significantly decreased in the shMCU + Cis group compared with the shControl + Cis group (Figure 6).

### 3.7 Ru360 cotreatment was associated with reduced cisplatin-induced injury in HEI-OC1 cells

**FIGURE 7:**
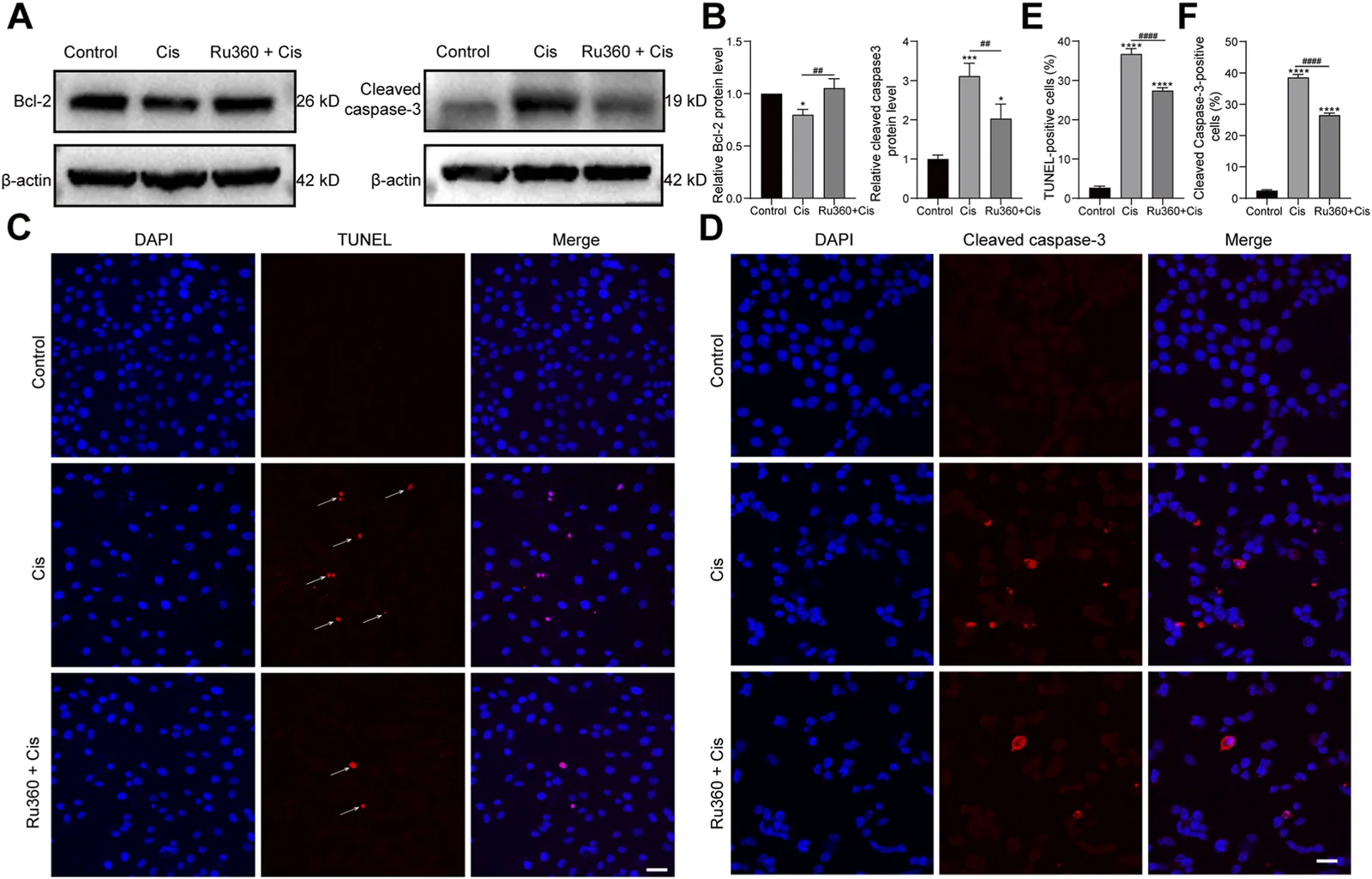
Ru360 cotreatment was associated with reduced cisplatin-induced injury in HEI-OC1 cells. **(A)** WB was utilized to assess the expression changes of apoptosis-related factors in HEI-OC1 cells treated with a combination of 10 μM Ru360 and 30 μM cisplatin. **(B)** Quantification of the WB results shown in panel **(A)**. **(C)** TUNEL assay was performed to investigate the effect of Ru360 on cellular apoptosis status. **(D)** Immunofluorescence was applied to detect changes in cleaved caspase-3 following Ru360 treatment. **(E)** Quantification of TUNEL-positive cells in panel H, expressed as a percentage of total cells. **(F)** Quantification of cleaved caspase-3-positive cells in panel I, expressed as a percentage of total cells. *p < 0.05, ***p < 0.001, ****p < 0.0001 compared to the Control group. ^##^p < 0.01, ^####^p < 0.0001 compared to the Cis group. n = 3. Scale bar for HEI-OC1 cells = 30 μm.

To identify a Ru360 concentration that did not independently compromise cell viability, HEI-OC1 cells were treated with 1–40 μM Ru360 for 24 h. No significant reduction was detected at 1, 5, or 10 μM, whereas the CCK-8 signal was significantly reduced at 20 and 40 μM (Supplementary Figure S1A). Therefore, 10 μM, the highest tested concentration without detectable cytotoxicity under these conditions, was selected for the subsequent cotreatment experiments. In parallel, MCU protein abundance was examined as an exploratory endpoint following 24 h of Ru360 exposure. Ru360 treatment was associated with decreased MCU protein abundance at 10, 20, and 40 μM, with the largest change observed at 10 μM, whereas the changes at 1 and 5 μM were not statistically significant (Supplementary Figures S1B,C). WB results revealed that the anti-apoptotic factor Bcl-2 was elevated, whereas the pro-apoptotic protein cleaved caspase-3 was decreased, with Ru360 cotreatment (Figures 7A,B). Compared with the cisplatin-alone group, the TUNEL assay showed that the percentage of TUNEL-positive cells was reduced in the Ru360 cotreatment group (Figures 7C,E). The trend of cleaved caspase-3 immunofluorescence staining was consistent with the WB results (Figures 7D,F).

### 3.8 Ru360 coadministration was associated with attenuation of cisplatin-induced auditory injury

**FIGURE 8:**
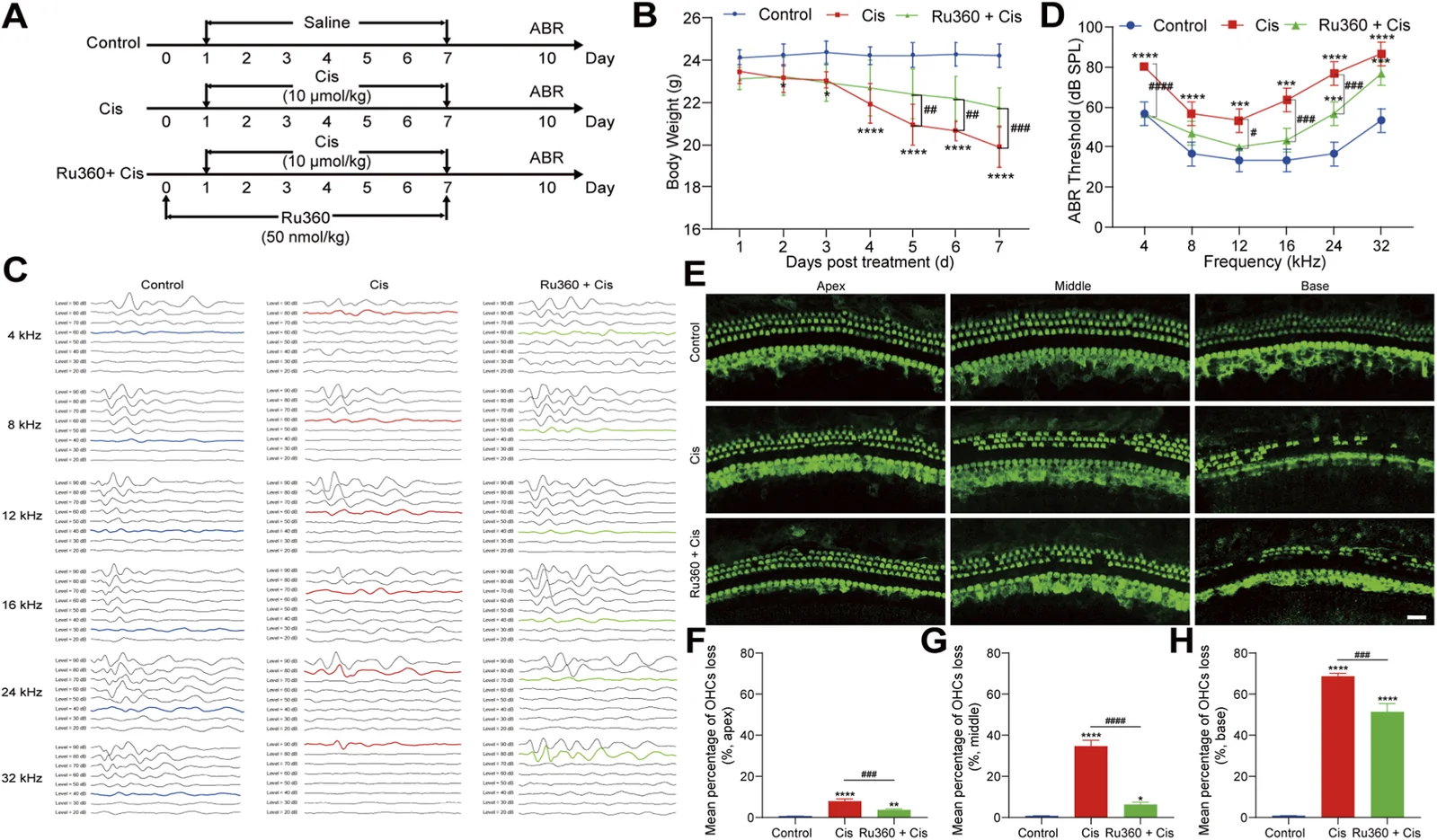
Ru360 coadministration was associated with attenuation of cisplatin-induced auditory injury. **(A)** Schematic of the *in vivo* experimental protocol. Mice were randomly divided into three groups: The Control, Cis, and Ru360 + Cis groups. Cisplatin (10 μmol/kg) was injected intraperitoneally on days 1–7, Ru360 (50 nmol/kg) on days 0–7, and saline served as the control; ABR was measured on day 10. **(B)** A line chart illustrated the changes in mouse body weight after drug administration. **(C)** ABR audiometry waveforms showed changes in hearing waveforms among different groups after drug administration. The Control group appeared normal, the Cis group showed disordered and deformed waveforms, while the Ru360 + Cis group showed improvement. The colored lines represented threshold waveforms. **(D)** A line chart quantitatively represents the hearing thresholds of mice. The results revealed that the hearing thresholds of the Control group were within the normal range, while those of the Cis group significantly increased at all frequencies. The Ru360 + Cis group showed partial improvement in hearing thresholds. **(E)** Immunofluorescence staining was used to assess the loss of hair cells in the cochlear basilar membrane of mice after Ru360 administration. In the Control group, no hair cell loss was observed. In the Cis group, outer hair cell loss occurred in the apical, middle, and basal turns, with the basal turn being the most severely affected, followed by the middle turn, and only individual outer hair cell loss in the apical turn. Compared to the Cis group, the application of Ru360 resulted in some relief from outer hair cell loss. **(F–H)** Quantitative statistical analysis of the proportion of lost outer hair cells in the apical, middle, and basal turns of the basilar membrane is presented in the bar charts. *p < 0.05, **p < 0.01, ***p < 0.001, ****p < 0.0001 compared to the Control group, ^#^p < 0.05, ^##^p < 0.01, ^###^p < 0.001, ^####^p < 0.0001 compared to the Cis group. n = 6. Scale bar for cochlear basilar membrane = 25 μm.

To determine whether Ru360 coadministration altered the severity of cisplatin-induced auditory injury *in vivo*, we used the treatment schedule shown in Figure 8A. Compared with the Cis group, the Ru360 + Cis group exhibited significantly less body weight loss and fewer outer hair cell losses, although the Cis group itself showed a marked decrease in body weight relative to the Control group (Figure 8B). ABR results revealed that the hearing thresholds of the Cis group under all stimuli were significantly elevated compared with the Control group, especially at high frequencies. With Ru360 cotreatment, the hearing thresholds were significantly decreased at 4, 12, 16, and 24 kHz, whereas no significant improvement was observed at 8 or 32 kHz (Figures 8C,D). In addition, the immunofluorescence showed that a large number of outer hair cells were lost after cisplatin administration in all cochlear turns, with the basal turn being the most vulnerable to cisplatin. Cotreatment with Ru360 significantly reduced the loss of outer hair cells (Figures 8E–H).

## 4 Discussion

Over the past decades, the exploration of MCU functions has predominantly focused on various diseases, whereas its role in hearing loss, particularly in cisplatin-induced ototoxicity, has remained relatively underexplored (Alves-Figueiredo et al., 2024; Bierhansl et al., 2024). The marked upregulation of MCU upon cisplatin exposure observed in this study suggests that MCU may play an important role in cisplatin-induced ototoxicity. Interestingly, compared with our previous work (Xu et al., 2024), the upregulation of MCU paralleled the decline in SERCA2 expression under cisplatin administration, suggesting a possible interplay between these two channels. Moreover, the fluorescence intensity of Mito-Tracker was decreased along with cisplatin insult, indicating mitochondrial function was impaired, although this measurement does not directly assess mitochondrial function or mitochondrial Ca^2+^. These findings add to the evidence on the role of MCU in the cochlea and indicate that MCU may represent a candidate target in cisplatin-induced ototoxicity.

Since the regulation of MCU by cisplatin is not direct, it becomes imperative to identify its upstream regulatory factors. Our prior studies have demonstrated that cisplatin triggers calcium disruption in ER and cytoplasm by modulating related calcium channels and then induces ER stress (Xu et al., 2024; Zhao et al., 2022). All of these events are intimately connected with mitochondria, which are important components of the calcium buffering system and have a direct connection with the ER through mitochondria-associated membranes (MAMs) (Szymański et al., 2017). The cisplatin-induced increase in MCU was partially reversed by chelating cytosolic calcium with BAPTA-AM and relieving ER stress with 4-PBA, indicating that calcium dysregulation and ER stress are important but not the sole drivers of MCU upregulation in this setting.

Given the importance of autophagy in mitochondrial dysfunction, the observed changes in Beclin1, LC3B-II, the LC3B-II/LC3B-I ratio, and p62 are compatible with altered autophagy-related processes during cochlear cell damage. However, measurements at individual time points cannot distinguish increased autophagosome formation from impaired lysosomal degradation and therefore do not establish the direction of autophagic flux. This distinction is important because autophagy has a context-dependent, double-edged role in the inner ear, with both cytoprotective and pro-death functions reported in cisplatin- and aminoglycoside-induced ototoxicity (Dai et al., 2024). In the present model, MCU knockdown under cisplatin exposure was accompanied by changes in autophagy-related markers, reduced apoptosis-related signaling, and improved cell survival. Because autophagic flux was not measured, these findings do not establish whether autophagy contributes to protection, promotes cell injury, or simply represents a secondary response to cell damage.

MCU knockdown alone did not affect baseline cell viability but markedly improved survival upon cisplatin exposure, indicating that loss of MCU is protective specifically under cisplatin stress rather than altering viability *per se*. SERCA2, the principal pump returning calcium from the cytoplasm to the ER, was upregulated following MCU knockdown irrespective of cisplatin exposure. It has been reported that MCU knockdown reduced the generation of ROS in mitochondria under hypoxic/reoxygenation conditions (Luongo et al., 2015). We therefore speculate that MCU may indirectly modulate SERCA2 by limiting ROS generation, with the resulting SERCA2 upregulation helping to restore calcium homeostasis; this hypothesis remains to be tested directly. In addition, the MAM components IP3R, GRP75, and VDAC1 were markedly decreased after MCU knockdown compared with the shControl + Cis group. These expression changes are compatible with altered ER–mitochondrial Ca^2+^ handling, but they do not establish that Ca^2+^ transfer from the ER to mitochondria was reduced. It has been reported that inhibition of VDAC1, a gatekeeper of mitochondria–cytosol calcium transport located in the outer mitochondrial membrane (OMM), alleviates ferroptosis and protects mitochondria, whereas its overexpression results in mitochondrial dysfunction and ROS accumulation (Shoshan-Barmatz et al., 2018; Zhou et al., 2023). In this work, VDAC1 was significantly decreased in the shMCU + Cis group compared to the shControl + Cis group. Together with the SERCA2 data, these changes may partly account for the cytoprotective effect of MCU knockdown.

Unbiased RNA-seq nominated autophagy, apoptosis, and the mTOR signaling pathway as the processes most strongly associated with the protective effect of MCU knockdown, with KEGG analysis specifically highlighting mTOR signaling. This provided a hypothesis-generating basis for the targeted validation described below.

Targeted validation confirmed activation of the PI3K/AKT/mTOR pathway upon MCU knockdown, as reflected by elevated p-PI3K, PDK1, p-AKT, and p-mTOR in the shMCU + Cis group. Considering the PI3K/AKT/mTOR pathway is an essential regulator of multiple cellular physiological processes, including proliferation, apoptosis, autophagy, and amino acid metabolism, its activation is generally recognized to suppress autophagy and confer prosurvival effects (Zou et al., 2020). Consistent with this, our study revealed that in cisplatin-treated groups, MCU knockdown led to significant reductions in Beclin1 and LC3B-II expression while increasing p62 expression, whereas the apoptotic executioner cleaved caspase-3 was downregulated. These coordinated changes establish an association among MCU knockdown, PI3K/AKT/mTOR-related signaling, autophagy-related markers, and reduced apoptosis-related signaling.

Lastly, Ru360, a widely used pharmacological inhibitor of MCU-mediated mitochondrial Ca^2+^ uptake, was used as an independent approach to examine the role of MCU-related signaling in cisplatin-treated cells (Southam et al., 2017). Ru360 cotreatment was associated with decreased cleaved caspase-3 and fewer TUNEL-positive cells, together with increased Bcl-2, resembling the changes observed after genetic knockdown. However, cochlear Ru360 exposure and direct target engagement were not measured, and possible Ru360–cisplatin interactions or effects on cisplatin availability, uptake, and cytotoxic activity were not tested. Consequently, the lower ABR thresholds and reduced OHC loss observed with Ru360 cotreatment cannot be attributed specifically to MCU inhibition. Cell-free interaction assays and measurements of cochlear platinum accumulation will be required to distinguish MCU-related effects from altered cisplatin availability or uptake. Two aspects of the Ru360 experiments merit further consideration. First, Ru360 acts primarily as a blocker of MCU channel activity rather than MCU expression or translation. Accordingly, the modest reduction in MCU protein observed after 24 h cannot be used as evidence of MCU inhibition and may represent a secondary response, an off-target effect, or experimental variability; its basis remains unresolved. Second, Ru360 has limited cell permeability, and its systemic delivery to the cochlea may be constrained by the blood-labyrinth barrier (Woods et al., 2019; Nyberg et al., 2019). Because cochlear Ru360 exposure was not quantified, the degree and distribution of drug exposure—and therefore cochlear target engagement—remain unknown. Direct measurement of MCU-dependent mitochondrial Ca^2+^ uptake and cochlear Ru360 exposure, together with the use of more cell-permeant MCU inhibitors (e.g., Ru265) and targeted delivery strategies, would strengthen the mechanistic interpretation of these findings.

In the *in vivo* experiments, the Ru360 + Cis group had lower ABR thresholds and less OHC loss than the Cis group. However, the ABR findings differed among frequencies. Thresholds were significantly lower at 4, 12, 16, and 24 kHz, whereas no significant difference was found at 8 or 32 kHz. Although Ru360 cotreatment was associated with less OHC loss in the basal turn, this histological difference was not accompanied by a significant change in the ABR threshold at 32 kHz. The remaining OHCs may not have retained normal function, and cisplatin-induced injury to other cochlear or neural structures may also have affected the ABR thresholds. Distortion-product otoacoustic emission (DPOAE) was not measured in this study, so we could not determine whether the surviving OHCs were functional. Future studies should combine DPOAE recordings with auditory brainstem response measurements and cochlear hair cell analysis, directly quantify cochlear drug exposure, and determine whether candidate MCU modulators preserve the antitumor efficacy of cisplatin. The absence of a significant difference at 8 kHz cannot be explained by the available data.

Several limitations constrain the mechanistic interpretation of these findings. First, mitochondrial Ca^2+^ was not directly measured; consequently, the present study cannot confirm that cisplatin increased mitochondrial Ca^2+^ or that MCU knockdown or Ru360 reduced mitochondrial Ca^2+^ accumulation. Future studies should directly assess mitochondrial Ca^2+^ using Rhod-2, mito-GCaMP, or CEPIA-based sensors. Second, autophagic flux was not quantified. Lysosomal inhibitors such as bafilomycin A1 or chloroquine, or tandem fluorescent LC3 reporters, are required to distinguish altered autophagosome formation from impaired degradation. Third, the causal contribution of PI3K/AKT/mTOR signaling was not tested. Pathway-blocking approaches, including LY294002, rapamycin, and complementary genetic strategies, are needed to determine whether this pathway is required for MCU knockdown-mediated protection. Fourth, most mechanistic experiments were performed in immortalized cochlear-derived HEI-OC1 cells, which lack stereociliary hair bundles and do not fully recapitulate mature cochlear hair cells. Although disruption of canonical hair-cell mechanotransduction channels is therefore unlikely to explain the BAPTA-AM findings, cisplatin uptake was not measured after BAPTA-AM treatment, and indirect effects of intracellular Ca^2+^ chelation on non-MET cisplatin uptake or intracellular handling cannot be excluded (More et al., 2010; Thomas et al., 2013). Validation in cochlear explants, primary hair cells, and *in vivo* genetic models is therefore required.

## 5 Conclusion

Taken together, MCU knockdown was associated with reduced cisplatin-induced injury and apoptosis-related signaling, accompanied by increased PI3K/AKT/mTOR signaling and alterations in autophagy-related markers. Ru360 treatment was likewise associated with partial protection *in vitro* and *in vivo*. These findings support MCU-related signaling as a candidate target in cisplatin ototoxicity, while direct mitochondrial Ca^2+^ measurements, autophagic-flux assays, pathway-intervention studies, and validation in mature hair-cell models are required to establish the underlying mechanism and therapeutic specificity.

## Data Availability

The datasets presented in this study can be found in online repositories. The names of the repository/repositories and accession number(s) can be found below: https://drive.google.com/drive/folders/1tSjEkQcVfuoEfInYK-uB-c82g44CU9De?usp=drive_link, https://drive.google.com/drive/folders/1tSjEkQcVfuoEfInYK-uB-c82g44CU9De?usp=drive_link.

## Supplementary material

SUPPLEMENTARY FIGURE S1
**(A)** CCK-8 was employed to examine the impact of various concentrations of Ru360 on the survival rate of HEI-OC1 cells after 24 h of treatment. **(B)** WB was used to detect the effect of different concentrations of Ru360 on MCU after treating HEI-OC1 cells for 24 h. **(C)** Quantification of the WB results shown in panel B. *p < 0.05, **p < 0.01, ***p < 0.001 compared to the Control group. n = 3.
